# *SREB,* a GATA Transcription Factor That Directs Disparate Fates in *Blastomyces dermatitidis* Including Morphogenesis and Siderophore Biosynthesis

**DOI:** 10.1371/journal.ppat.1000846

**Published:** 2010-04-01

**Authors:** Gregory M. Gauthier, Thomas D. Sullivan, Sergio S. Gallardo, T. Tristan Brandhorst, Amber J. Vanden Wymelenberg, Christina A. Cuomo, Garret Suen, Cameron R. Currie, Bruce S. Klein

**Affiliations:** 1 Department of Medicine, University of Wisconsin, Madison, Wisconsin, United States of America; 2 Department of Pediatrics, University of Wisconsin, Madison, Wisconsin, United States of America; 3 Department of Medical Microbiology & Immunology, University of Wisconsin, Madison, Wisconsin, United States of America; 4 Broad Institute of MIT and Harvard, Cambridge, Massachusetts, United States of America; 5 Department of Bacteriology, University of Wisconsin, Madison, Wisconsin, United States of America; University of Birmingham, United Kingdom

## Abstract

*Blastomyces dermatitidis* belongs to a group of human pathogenic fungi that exhibit thermal dimorphism. At 22°C, these fungi grow as mold that produce conidia or infectious particles, whereas at 37°C they convert to budding yeast. The ability to switch between these forms is essential for virulence in mammals and may enable these organisms to survive in the soil. To identify genes that regulate this phase transition, we used *Agrobacterium tumefaciens* to mutagenize *B. dermatitidis* conidia and screened transformants for defects in morphogenesis. We found that the GATA transcription factor *SREB* governs multiple fates in *B. dermatitidis*: phase transition from yeast to mold, cell growth at 22°C, and biosynthesis of siderophores under iron-replete conditions. Insertional and null mutants fail to convert to mold, do not accumulate significant biomass at 22°C, and are unable to suppress siderophore biosynthesis under iron-replete conditions. The defect in morphogenesis in the *SREB* mutant was independent of exogenous iron concentration, suggesting that *SREB* promotes the phase transition by altering the expression of genes that are unrelated to siderophore biosynthesis. Using bioinformatic and gene expression analyses, we identified candidate genes with upstream GATA sites whose expression is altered in the null mutant that may be direct or indirect targets of *SREB* and promote the phase transition. We conclude that *SREB* functions as a transcription factor that promotes morphogenesis and regulates siderophore biosynthesis. To our knowledge, this is the first gene identified that promotes the conversion from yeast to mold in the dimorphic fungi, and may shed light on environmental persistence of these pathogens.

## Author Summary

The dimorphic fungi are the most common cause of invasive fungal disease worldwide. In the soil, these fungi grow as mold that produce infectious spores; when inhaled into the warmer lungs of a mammalian host, the spores convert into yeast, which cause infection. The change in shape between mold and yeast is a crucial event in the lifecycle of these fungi. The molecular regulation of this morphologic switch, or phase transition, is poorly understood. The goal of our research was to identify and characterize novel gene(s) that govern the phase transition in dimorphic fungi using *Blastomyces dermatitidis* as a model organism. Using insertional mutagenesis, we identified a gene, *SREB,* which encodes a transcription factor that affects phase transition and regulates the production of iron-gathering molecules or siderophores. When *SREB* is deleted, *B. dermatitidis* fails to complete the conversion from yeast to mold, grows poorly at environmental temperature, has yellow-orange colony pigmentation, and cannot properly repress the biosynthesis of siderophores. We also identified two types of siderophores produced by *B. dermatitidis.* To our knowledge, *SREB* is the first gene identified that promotes the conversion from yeast to mold, a process important for survival in the environment and generation of infectious spores.

## Introduction

The endemic dimorphic fungi are comprised of seven ascomycetes that include Blastomyces dermatitidis, Histoplasma capsulatum, Coccidioides immitis, Coccidioides posadasii, Paracoccidioides brasiliensis, Sporothrix schenckii, and Penicillium marneffei. These fungi possess the unique ability to switch between two different morphologies, yeast and mold, in response to external stimuli [1]. In nature, they grow as mycelia that produce conidia, which are the infectious particles; when aerosolized spores are inhaled into the warmer lungs of a mammalian host, they convert into pathogenic yeast and cause necrotizing infection [1]. The dimorphic fungi collectively are the most common cause of invasive fungal disease worldwide and account for several million infections each year [2]. Unlike opportunistic fungi, such as Cryptococcus or Aspergillus, the dimorphic fungi can infect both immunocompetent and immunocompromised hosts [3]–[5]. The size of the inhaled inoculum and the integrity of the cell-mediated immune system influence the extent and severity of infection [1],[3]. Clinical manifestations range from asymptomatic infection to symptomatic disease and include pneumonia, acute respiratory distress syndrome, and disseminated disease involving multiple organ systems [1],[3].

The ability of the dimorphic fungi to switch between the two different morphologies is crucial for pathogenesis. Although temperature is postulated to be the major stimulus that induces phase transition, other stimuli, including carbon dioxide tension, steroid hormones, and oxidative stress influence this morphologic switch [1], [6]–[9]. Phase transition is a complex process that involves the coordinated expression and repression of many genes in response to external stimuli, which alters cell wall composition, metabolism, intracellular signaling, and morphology [10]–[13]. The identification of *DRK1* (dimorphism-regulating kinase-1) in *B. dermatitidis* and *H. capsulatum* offered strong genetic evidence that phase transition is required for pathogenicity [10]. *DRK1* functions as a global regulator and has pleotropic effects on the cell, controlling morphogenesis, cell wall composition, sporulation, expression of yeast-phase specific genes, and virulence. *DRK1* null mutants remain locked in the mycelial phase, fail to sporulate or express the essential virulence factors *BAD1* (*Blastomyces* adhesin-1 in *B. dermatitidis*) and *CBP1* (Calcium binding protein-1 in *H. capsulatum*), and are avirulent in a murine model of infection [10]. Three additional genes, *RYP1*, *RYP2*, and *RYP3*, have been described that regulate morphogenesis in *H. capsulatum*. Silencing the expression of *RYP1, 2 or 3* results in hyphal growth at 37°C and inappropriate sporulation [12],[13].

The goal of this study was to identify and characterize additional genes that regulate the phase transition in dimorphic fungi, using *B. dermatitidis* as a model system. While progress has been made in identifying genes that regulate the morphological transition from mold to yeast, to our knowledge, no genes have been identified that regulate the switch in the other direction in the dimorphic fungi – that is, from the yeast to mold form. The mold form is believed to be required for the growth and survival of the dimorphic fungi in the environment by enabling propagation in soil and transmission to humans through the generation of conidia. Herein, we describe a gene, *SREB*, identified through insertional mutagenesis, which impacts multiple disparate fates in *B. dermatitidis,* including the phase transition of yeast to mold, cell growth at 22°C, and the biosynthesis of siderophores.

## Results

### Insertional mutagenesis and identification of *SREB*

**Figure 1:**
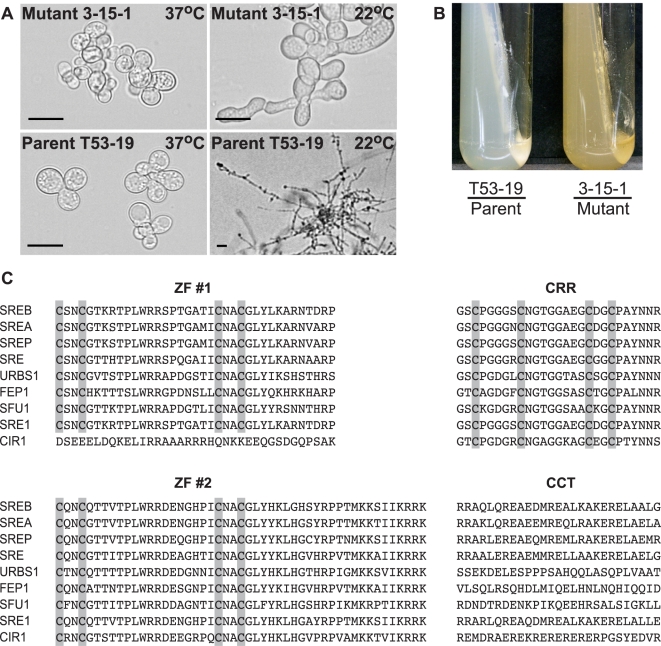
Phenotype of insertional mutant 3-15-1 and conserved motifs in SREB. (**A**) Insertional mutant 3-15-1 failed to convert from yeast to mold after 14 days of incubation at 22°C. Parent strain T53-19 converted from yeast to mycelia within 7 days of shifting the temperature from 37°C to 22°C (Scale bar equals 20 µm). (**B**) Mutant 3-15-1 developed a yellow pigmentation and discolored the surrounding 7H10 medium, which contains 150 µM FeSO_4_. In contrast, T53-19 grew as white-colored yeast and did not pigment the medium. (**C**) The predicted amino acid sequence of *B. dermatitidis* SREB is aligned with *A. nidulans* SREA, *P. chrysogenum* SREP, *N. crassa* SRE, *U. maydis* URBS1, *S. pombe* FEP1, *C. albicans* SFU1, *H. capsulatum* SRE1, and *C. neoformans* CIR1. SREB contained several conserved domains including two zinc fingers (ZF #1, ZF #2) separated by a cysteine-rich region (CRR) and a conserved C-terminus (CCT) with a predicted coiled-coil domain. Conserved cysteine residues in ZF #1, ZF #2, and CRR are highlighted. ClustalW (1.81) was used to align the amino acid sequences. GeneBank accession numbers include AAD25328 (SREA), AAC49628 (SREP), AAC64946 (SRE), AAB05617 (URBS1), AAM29187 (FEP1), XP_723364 (SFU1), ABY66603 (SRE1), and ABG21303 (CIR1).

*Agrobacterium tumefaciens*-mediated DNA transfer was used to mutagenize haploid, uninucleate conidia of *B. dermatitidis* strain T53-19. Following selection with hygromycin, 22,000 transformants were visually screened by light microscopy for morphologic alterations including growth as hyphae or pseudohyphae at 37°C or as yeast at 22°C. In this study, one of the mutants identified by the screen, 3-15-1, was characterized in detail. This mutant, unlike the parent strain, was pigmented yellow and failed to complete the conversion from yeast to mold (**Figure 1A, 1B**). Southern blot hybridization demonstrated a single site of insertion (**Figure S1**).

The genomic DNA flanking the insert in 3-15-1 was amplified using adapter PCR, sequenced, and analyzed using a BLASTn search against the genome sequence of *B. dermatitidis* strain 26199. No rearrangements or deletions were identified in the DNA flanking the insert. Additional BLAST analyses indicated that the insert interrupted a region 692 base-pairs (bp) upstream of a predicted open reading frame with nucleotide homology to *Pencillium chrysogenum SREP,* which encodes a GATA transcription factor that regulates the biosynthesis of siderophores [14]. We named this homolog *SREB* (siderophore biosynthesis repressor in *Blastomyces*) in *B. dermatitidis*.

### *SREB* sequence analysis

FGENESH analysis of the nucleotide sequence predicted that *SREB* contained a 1909 nucleotide (nt) coding region interrupted by two short introns (88 and 74 nt). Each intron was located in a zinc-finger coding region and contained the expected 5′-splice donor (GTNNGT) and 3′-splice acceptor (pyrimidine-AG) sequences [15]. The length, location, and number of introns interrupting the open reading frame were conserved among *B. dermatitidis SREB*, *H. capsulatum SRE1*, *A. nidulans SREA*, and *N. crassa SRE* [16]–[18]. The *SREB* coding region was predicted to encode a 636 amino acid protein.

The predicted amino acid sequence of *SREB* had homology to siderophore biosynthesis repressors in other fungi including *Aspergillus nidulans* SREA, *Penicillum chrysogenum* SREP, *Neurospora crassa* SRE, *Ustilago maydis* URBS1, *Schizosaccharomyces pombe* FEP1, *Candida albicans* SFU1, *Cryptococcus neoformans* CIR1, and *Histoplasma capsulatum* SRE1 (**Figure 1C**) [14], [16]–[22]. SREB contained several conserved domains characteristic of GATA transcription factors that regulate iron assimilation, including two zinc finger motifs separated by a cysteine-rich region (CRR) and a C-terminus predicted to have a coiled-coil domain (**Figure 1C**) [17],[23]. With the exception of *C. neoformans* CIR1, fungal GATA transcription factors that regulate the acquisition of iron contain two zinc fingers [22]. This zinc finger arrangement is unique because most GATA transcription factors in fungi contain only one zinc finger [17]. The cysteine residues in each zinc finger of SREB were arranged in a conserved class IV motif, Cys-X_2_-Cys-X_17_-Cys-X_2_-Cys [24]. The cysteine-rich region contained four conserved cysteine residues, which have been demonstrated to coordinate the binding of iron in *H. capsulatum* [16].

### Phenotypes of insertional mutant 3-15-1

Mutant 3-15-1 failed to convert from yeast to mycelia or produce conidia following a shift in incubation temperature from 37°C to 22°C (**Figure 1A**). In contrast, the parent strain T53-19 converted to mycelia when grown at 22°C and produced conidia. Mutant 3-15-1 accumulated little biomass at 22°C, but remained viable (as measured by the exclusion of 0.2% eosin stain), and converted to normal yeast morphology when the incubation temperature was shifted back to 37°C (data not shown).

**Figure 2:**
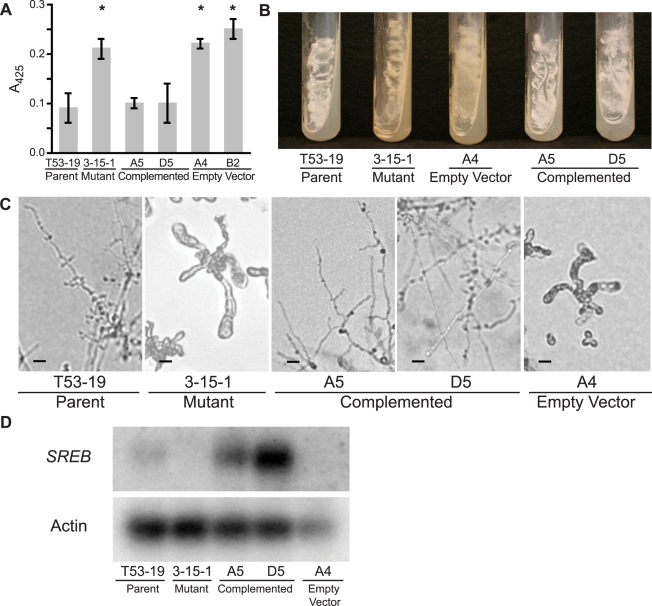
Complementation of insertional mutant 3-15-1. (**A**) Under iron-replete conditions (10 µM FeSO_4_), mutant 3-15-1 and empty vector controls fail to suppress siderophore biosynthesis as demonstrated by a 2.5–3.0 fold higher absorbance at 425 nm compared to parental strain T53-19 and complemented strains A5 and D5 (p<0.01 as indicated by asterisk). Data from three independent experiments were analyzed. Siderophore production was measured using the ferric perchlorate assay. (**B**) Mutant 3-15-1 and empty vector control A4 were pigmented yellow and discolored the surrounding media when grown on 7H10 medium, which contains 150 µM FeSO_4_. In contrast, parental control T53-19 and complemented strains A5 and D5 were colored white and did not pigment the medium. All strains were incubated for 14 days at 37°C. (**C**) Mutant 3-15-1 and empty vector control A4 fail to convert to mycelia upon shifting the incubation temperature from 37°C to 22°C. Similar to the wild-type isolate, complemented strains A5 and D5 grew as mycelia at 22°C incubation. (**D**) Northern blot hybridization demonstrated reduction in transcript abundance in the insertional mutant when compared to the parent strain. Complemented strains A5 and D5 overexpress *SREB*. Transformation of 3-15-1 with an empty vector failed to restore transcript abundance in strain A4.

The yellow-orange pigmentation of mutant 3-15-1 and the predicted amino acid sequence suggested that *SREB* functioned as a repressor of siderophore biosynthesis. Deletions of *SREB* homologs in *P. chrysogenum* (*SREP*), *A. nidulans* (*SREA*), and *N. crassa* (*SRE*) produce similar discoloration [14],[17],[18]. To assess for the dysregulation of siderophore biosynthesis in the insertion mutant, we used a colorimetric assay to detect the production of hydroxymate-type sideophores in culture supernatants [25]. Under iron-poor conditions, both T53-19 and 3-15-1 produced an abundance of siderophores as measured by this assay (data not shown). Under iron-replete conditions, mutant 3-15-1 continued to produce siderophores, whereas parent strain T53-19 repressed siderophore biosynthesis (**Figure 2A**).

### Complementation of mutant 3-15-1

To determine if the mutant phenotype was from altered expression of *SREB*, and not due to another mutation incurred during insertional mutagenesis, we set out to complement the mutant phenotype. Insertional mutant 3-15-1 was re-transformed via *A. tumefaciens* to provide an intact gene copy of *SREB* and its endogenous promoter. Complemented strains A5 and D5 grew as white colonies that did not discolor the medium, suppressed siderophore production under iron-replete conditions (10 µM FeSO_4_), and converted fully to mycelia when grown at a temperature of 22°C (**Figure 2A-C**). Retransformation of 3-15-1 with a vector lacking *SREB* did not complement the mutant phenotype (empty vector strain) (**Figure 2A-C**). Whereas Northern analysis demonstrated a reduction in the abundance of *SREB* transcript in mutant 3-15-1 compared to the parental strain, message levels were overexpressed in both complemented strains (**Figure 2D**). Thus, complementation reversed the mutant's phenotypic defects, supporting the idea that the insert was responsible for the dysregulation of siderophore biosynthesis and the alteration in morphogenesis.

### Disruption of *SREB*

**Figure 3:**
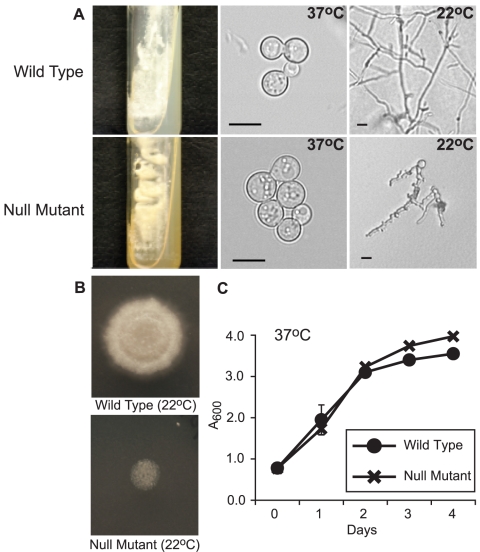
Phenotype of the *SREB* null mutant. (**A**) When grown on 7H10 medium containing 150 µM FeSO_4_, the null mutant (*SREB*Δ) grew as yellow-orange pigmented colonies that discolored the medium (37°C; 15 days incubation). In contrast the wild-type strain (ATCC 26199) grew as white colored yeast and did not pigment the medium. At 37°C, *SREB*Δ and wild-type isolates grew as budding yeast. Following a shift in temperature from 37°C to 22°C, *SREB*Δ failed to complete the conversion from yeast to mycelia (17 days; HMM medium). (**B**) The null mutant (*SREB*Δ) does not accumulate significant biomass or expand by radial growth when compared to the wild-type isolate. For each strain, 2.5×10^4^ yeast were spotted on HMM medium and incubated at 22°C for 14 days. (**C**) The null mutant (*SREB*Δ) and wild-type isolates have a similar growth rate when they are cultured as yeast at 37°C incubation. Culture density was measured in triplicate at A_600_. Cultures were grown in liquid HMM supplemented with 10 uM FeSO_4_. The data were from two independent experiments.

To confirm that *SREB* represses the biosynthesis of siderophores and affects morphogenesis in *B. dermatitidis*, we disrupted this gene in wild-type isolate 26199 using homologous recombination. To minimize the probability that the phenotype observed in mutant 3-15-1 was unique to strain T53-19, we used a different *B. dermatitidis* strain, 26199, to generate a null mutant. The rate of allelic replacement was 0.04% (1/2670). The null mutant, *SREB*Δ, grew as yellow-pigmented colonies that discolored the surrounding medium and failed to properly repress siderophore biosynthesis when iron was abundant (**Figure 3A** **,** **5B**). The intensity of pigmentation was dependant on exogenous iron and independent of temperature (37°C vs. 22°C) (data not shown). In contrast, the parent strain grew as white-colored yeast and repressed the production of siderophores under iron-replete conditions as measured by the ferric perchlorate assay (**Figure 3A** **,** **5B**). *SREB*Δ failed to complete the yeast-to-mold phase transition following a shift in temperature from 37°C to 22°C, did not exhibit radial growth, and accumulated little biomass at 22°C (**Figure 3A, 3B**). The defect in phase transition persisted during prolonged incubation (>14 days) at 22°C; however, a few hyphal strands would develop and could only be observed by light microscopy. Similar to insertional mutant 3-15-1, *SREB*Δ remained viable at 22°C (as measured by 0.2% eosin exclusion) and converted back to yeast following a shift in temperature from 22°C to 37°C (data not shown). In the yeast form, the *SREB*Δ mutant grew at the same rate as the parent strain (**Figure 3C**). The morphologic defect at 22°C was independent of exogenous iron concentrations (data not shown).

**Figure 4:**
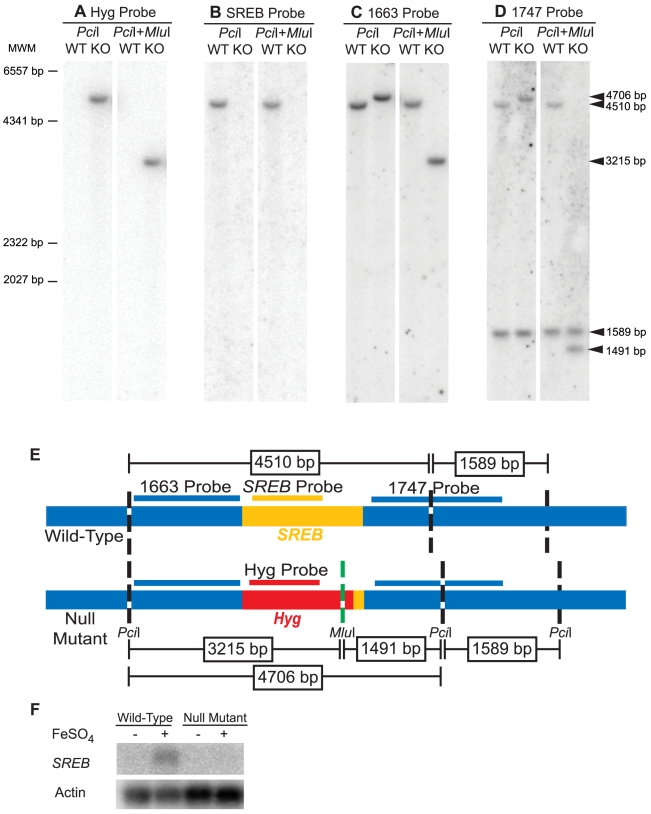
Southern and Northern blot analyses of the *SREB* null mutant. (**A-D**) Southern analysis of 26199 wild-type (WT) and null mutant *SREB*Δ (KO). Genomic DNA from WT and KO were digested with *Pci*I alone or in combination with *Mlu*I. The hygromycin resistance cassette, but not *SREB*, contains a *Mlu*I restriction site. The blots were probed against hygromycin, Hyg probe (A); *SREB*, SREB probe (B); the 5′ flank, 1663 probe (C); and 3′ flank, 1747 probe (D). Arrows indicate hybridizing fragments and dashes depict molecular weight markers (MWM). The Hyg and SREB probes failed to hybridize to digested WT or KO DNA, respectively (A, B). The 1663 and 1747 probes, which flank *SREB*, gave the expected size restriction fragments, which indicated a clean replacement of *SREB* sequence with the hygromycin resistance cassette (C, D). (**E**) Schematic illustrating the location of the restriction sites in the wild-type and null mutant (*SREB*Δ), hybridization sites for probes *Hyg*, *SREB*, 1663, and 1747, and expected size of the restriction fragments. Yellow, red, and blue indicate the *SREB* coding region, hygromycin resistance cassette, and sequence flanking *SREB*, respectively. (**F**) Northern analysis of 26199 wild-type and *SREB*-null mutant when grown under iron-poor (−) and iron-replete (+; 10 µM FeSO_4_) conditions. *SREB* transcript is detectable in the wild-type isolate under iron-replete conditions and absent in *SREB*Δ.

Analysis of the null mutant by PCR indicated disruption of *SREB* and the absence of any deletion or rearrangment of the genomic DNA flanking the transgene (data not shown). Southern blot analyses demonstrated replacement of *SREB* with a hygromycin resistance cassette and the absence of additional deletions in the genomic DNA flanking the transgene in *SREB*Δ (**Figure 4A-E**). Northern analysis demonstrated the loss of *SREB* transcript in *SREB*Δ (**Figure 4F**).

### Complementation of *SREB*

**Figure 5:**
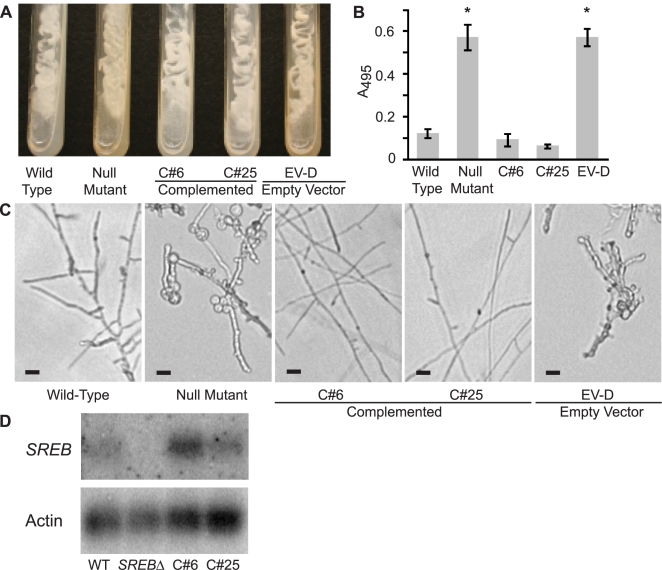
Complementation of the *SREB* null mutant. (**A**) Complemented strains C#6 and C#25 grew as white-colored yeast, similar to the wild-type isolate, on 7H10 slants (150 µM FeSO_4_). In contrast, the null mutant (*SREB*Δ) and empty vector strain EV-D grew as yellow-colored yeast and discolored the medium. Cultures were grown at 37°C for 14 days. (**B**) Complemented strains C#6 and C#25 were able to repress the biosynthesis of siderophores when grown-under iron-replete (10 µM FeSO_4_) conditions as measured by the ferric perchlorate assay. In contrast, the null mutant and empty vector (EV-D) continued to produce siderophores (p<0.01). Data from three independent experiments were analyzed. (**C**) Wild-type and complemented strains C#6 and C#25 convert to mycelia within 17 days of incubation at 22°C. In contrast, the null mutant and empty vector EV-D strains fail to complete the conversion to mycelia. (**D**) Northern blot hybridization demonstrated restoration of *SREB* transcript abundance in complemented strains C#6 and C#25, when compared to the null mutant.

To confirm the phenotype in *SREB*Δ was due to disruption of the siderophore biosynthesis repressor gene, we re-transformed the null mutant using *A. tumefaciens* to insert a copy of *SREB*. Complemented strains grew as white-colored colonies and properly suppressed the biosynthesis of siderophores when iron was abundant (**Figure 5A, 5B**). Following a temperature shift from 37°C to 22°C, complemented yeast strains converted to mold (**Figure 5C**). This conversion was slower in the complemented strains (14–17 days) when compared to the wild-type isolate (<7 days) (data not shown). The complemented strains underwent radial growth at 22°C; however, colony expansion was less than the wild-type isolate (data not shown). Prolonged incubation did not result in catch-up growth. Analysis of transcript abundance demonstrated restoration of message levels in C#25 and overexpression in C#6 when compared to wild-type and *SREB*Δ strains (**Figure 5D**).

### Expression of *SREB* and regulation of siderophore biosynthesis

To test if the expression of *SREB* was influenced by the concentration of exogeneous iron, we grew wild-type *B. dermatitidis* strain 26199 under iron-poor and –replete conditions. Northern blot analysis demonstrated that the expression of *SREB* was increased during conditions of iron abundance and repressed when iron was limited (**Figure 4F**).

**Figure 6:**
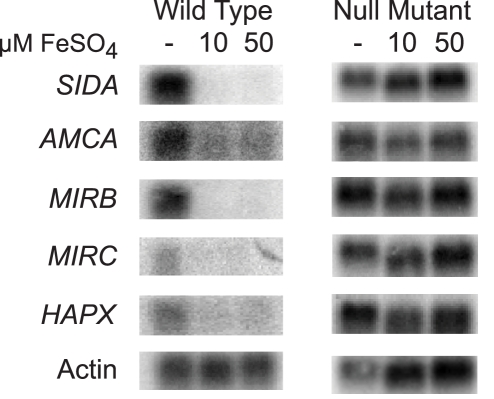
Northern analysis of candidate genes in the *SREB* regulon involved with siderophore biosynthesis and uptake. Wild-type *B. dermatitidis* strain 26199 upregulated the expression of *SIDA*, *AMCA*, *MIRB, MIRC,* and *HAPX* when grown in iron-poor medium (—); expression of these genes was repressed when iron was abundant (10 and 50 µM FeSO_4_). Deletion of *SREB* resulted in de-repression of *SIDA*, *AMCA, MIRB, MIRC,* and *HAPX* under iron-replete conditions.

In fungi, the expression of genes that encode proteins involved with iron assimilation are often co-expressed or -repressed when iron is limited or abundant, respectively. To investigate whether this was also true in *B. dermatitidis*, we analyzed the expression of several genes in response to exogenous iron. Under iron-poor conditions, *B. dermatitidis* wild-type strain 26199 induced the expression of genes involved in the biosynthesis of siderophores (*SIDA*), transport of ornithine from the mitochondria into the cytosol (*AMCA*), uptake of siderophores (*MIRB*, *MIRC*), and a bZIP transcription factor (*HAPX*) (**Figure 6**). Conversely, these genes were repressed when iron was abundant (**Figure 6**). The disruption of *SREB* de-repressed the expression of each of these genes. Thus, *SREB* regulates genes involved in siderophore biosynthesis and uptake in *B. dermatitidis* (**Figure 6**).

### Identification and characterization of *B. dermatitidis* siderophores

**Figure 7:**
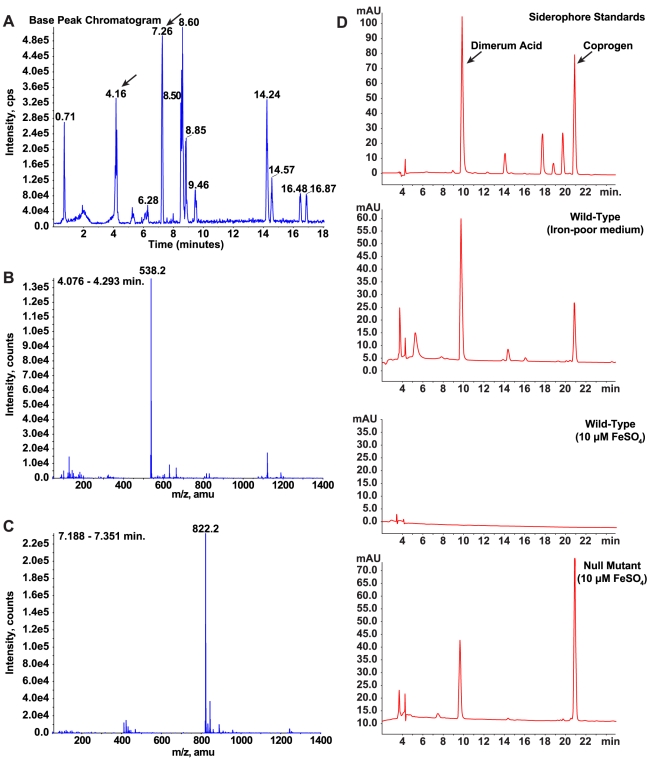
Identification and characterization of siderophores in *B. dermatitidis*. (**A-C**) Culture supernatant harvested from wild-type *B. dermatitidis* grown under iron-poor conditions was applied to a column packed with XAD-2 resin. Following a water wash, compounds bound to the resin were eluted with methanol and analyzed by LC/MS. The chromatogram revealed the presence of several compounds in the eluate (A). Analysis of these compounds using mass spectroscopy revealed that two of the peaks (4.16 and 7.26 minutes) had masses consistent with dimerum acid (538.2) and coprogen (822.2), respectively (B, C). (**D**) Culture supernatants harvested from wild-type *B. dermatitidis* grown under iron-poor and iron-replete conditions were compared to siderophore standards using HPLC. During conditions of iron-limitation, the wild-type isolate produced and secreted dimerum acid and coprogen. When iron was abundant, no siderophores were detected. In contrast, the null mutant *SREB*Δ produced and secreted dimerum acid and coprogen under iron-replete conditions.

To further characterize the regulatory role of *SREB* on siderophore biosynthesis, we used LC/MS and reverse-phase HPLC to identify the specific type(s) of siderophores secreted by *B. dermatitidis* wild-type and null mutant yeast cells. Starting with wild-type cells grown under iron-limited conditions, siderophores from culture supernatant were isolated using column chromatography. Mass spectroscopy of the eluate showed two large peaks at 4.16 and 7.26 minutes with molecular weights of 538.2 and 822.2 that correspond to dimerum acid and coprogen, respectively (**Figure 7A-C**). Reverse-phase HPLC of the eluate and comparison of retention times to siderophore standards confirmed the identities of these siderophores (**Figure 7D**). Under iron-replete conditions, wild-type *B. dermatitidis* repressed the biosynthesis of dimerum acid and coprogen (**Figure 7D**). In contrast, the null mutant continued to produce both siderophores (**Figure 7D**).

### Identification of candidate genes that promote the phase transition

To identify candidate genes regulated by *SREB* that may promote the phase transition, we first used MAST analysis to search the *Blastomyces* genome for GATA transcription factor-binding motifs in intergenic regions located ≤2000 bp upstream of predicted genes. Our initial search for the classic GATA transcription factor-binding motif, HGATAR, revealed the presence of this motif upstream of nearly all *B. dermatitidis* genes. This finding is similar to Schrettl et al., who found widespread distribution of this motif in *Aspergillus fumigatus* [26].

An extended version of the HGATAR motif, ATC-w-gAta-a, has been recently described and was demonstrated to occur at a 5.4-fold higher frequency in the promoter of genes regulated by *A. fumigatus SREA*, an *SREB* homolog, when compared to the entire *A. fumigatus* genome [26]. We revised our strategy and searched for this extended motif in the promoter of genes in the *B. dermatitidis* genome. We identified a total of 1,213 genes with at least one of the following motifs located ≤2 kb upstream of the start codon: ATC-(A/T)-GATA-(A/G), ATC-(A/T)-GATA-(T/C), ATC-(A/T)-GATT-A, ATC-(A/T)-GATC-A, ATC-A-GATG-A, ATC-C-GATA-A, and ATC-A-AATA-A. This gene-set included genes involved in siderophore biosynthesis and uptake (i.e. *SIDA, MIRB, AMCA*). Two or more upstream GATA motifs were present in 232 (19.1%) in the gene-set. Hwang and colleagues identified the motif (G/A)-ATC-(A/T)-GATA-A upstream of siderophore biosynthesis and transport genes regulated by *SID1* in *H. capsulatum* [27]. We found this longer motif upstream of 271 (22.3%) of our 1,213 MAST-identified genes; however, *MIRB* and *MIRC*, both involved in siderophore uptake, lacked the motif.

To classify the 1,213 candidate genes into functional categories and facilitate further analysis, we annotated the predicted protein products of these genes as well as the complete *B. dermatitidis* predicted proteome against the eukaryotic orthologous groups (KOG) database. The results, shown in **Table 1**, indicate that the KOG-annotated GATA-containing genes fall into many categories of gene function (i.e. transcription, RNA metabolism, signal transduction, cell remodeling and metabolism). The frequency of KOG-annotated genes with upstream GATA motifs within a particular KOG category was compared to the frequency of genes in the same KOG category within all KOG-annotated genes in the *B. dermatitidis* genome. Three KOG categories were significantly over-represented in the candidate gene-set harboring GATA sites: amino acid transport and metabolism (KOG code E), secondary metabolites biosynthesis, transport and catabolism (KOG code Q), and lipid transport and metabolism (KOG code I) (Table 1 and Table S1). This suggests that these cellular process pathways may be important for *SREB* regulation, although it does not exclude a role for the GATA-containing genes in other KOG groupings.

**Table 1:** Enrichment of GATA-containing genes in *B. dermatitidis* according to KOG category*.

KOG Category	KOG Code	GATA Genes	All Genes
**Information storage and processing**			
Translation, ribosomal structure and biogenesis	J	33 (6.33%)	368 (5.9%)
RNA processing and modification	A	21 (4.03%)	282 (4.52%)
Transcription	K	30 (5.76%)	433 (6.94%)
Replication, recombination and repair	L	20 (3.84%)	217 (3.48%)
Chromatin structure and dynamics	B	12 (2.3%)	124 (1.99%)
**Cellular processes and signaling**			
Cell cycle control, cell division, chromosome partitioning	D	17 (3.26%)	208 (3.33%)
Nuclear structure	Y	2 (0.38%)	42 (0.67%)
Defense mechanisms	V	4 (0.77%)	39 (0.63%)
Signal transduction mechanisms	T	42 (8.06%)	524 (8.4%)
Cell wall/membrane/envelope biogenesis	M	9 (1.73%)	74 (1.19%)
Cell motility	N	0 (0%)	5 (0.08%)
Cytoskeleton	Z	8 (1.54%)^†^	261 (4.18%)
Extracellular structures	W	1 (0.19%)	10 (0.16%)
Intracellular trafficking, secretion, and vesicular transport	U	28 (5.37%)	471 (7.55%)
Posttranslational modification, protein turnover, chaperones	O	41 (7.87%)	499 (8%)
**Metabolism**			
Energy production and conversion	C	23 (4.41%)	274 (4.39%)
Carbohydrate transport and metabolism	G	12 (2.3%)	210 (3.37%)
Amino acid transport and metabolism	E	36 (6.91%)•	264 (4.23%)
Nucleotide transport and metabolism	F	9 (1.73%)	77 (1.23%)
Coenzyme transport and metabolism	H	4 (0.77%)	92 (1.47%)
Lipid transport and metabolism	I	35 (6.72%)•	284 (4.55%)
Inorganic ion transport and metabolism	P	9 (1.73%)	162 (2.6%)
Secondary metabolites biosynthesis, transport & catabolism	Q	22 (4.22%)•	168 (2.69%)
**Poorly characterized**			
General function prediction only	R	72 (13.82%)	784 (12.57%)
Function unknown	S	31 (5.95%)	366 (5.87%)
**Total KOG-annotated Proteins**		**521**	**6238**

*: The predicted proteins of 1,213 candidate genes identified by MAST analysis were annotated using the KOG database and compared to all 6,238 KOG-annotated genes in the genome of *B. dermatitidis* strain SLH14081. Not all 1,213 genes could be assigned to a KOG category, particularly genes encoding “predicted proteins” or “conserved hypothetical proteins.” The number of genes per KOG category and their percentage among the KOG-annotated proteins in that group are shown for both sets of genes. A two-tailed Fisher's Exact Test was used to determine if a particular KOG category in the GATA transcription factor gene set was over- or under-represented with respect to all genes in the genome. Over-represented categories are denoted with a (•) at a *P*-value<0.05 and under-represented categories are denoted with a (**†**) at a *P*-value<0.05.

**Figure 8:**
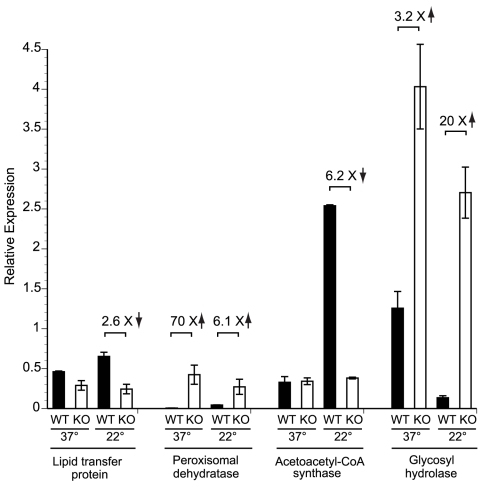
Expression of candidate genes in the *SREB* regulon at 37°C and 22°C. Quantitative RT-PCR (qRT-PCR) was performed to validate the expression of a subset of genes identified by bioinformatic and gene expression analyses. RNA was extracted from the wild-type (WT) strain and null mutant (KO) at 37°C and at 22°C (48 hours following the downward temperature shift). Deletion of *SREB* resulted in altered expression of genes involved with lipid metabolism and transport (lipid transfer protein, peroxisomal dehydratase, acetoacetyl-CoA synthase) as well as a glycosyl hydrolase that is predicted to have endo-1,3-β-glucanase activity. Data was generated from two biological replicates. qRT-PCR fluorescence was normalized to α-tubulin.

In a complimentary approach to identify genes that may be regulated by *SREB*, we performed a preliminary microarray analysis. Using an expression array with 70-mer oligonucleotides representing the 10,567 open reading frames of *B. dermatitidis* strain 26199, we used two-color spotted analysis to compare isogenic wild-type vs. *SREB*Δ at 37°C and at 22°C 48 hours after the temperature shift downward (data not shown). At least 38 of the genes identified by MAST analysis were differentially expressed (increased or decreased by ≥2-fold), including seven genes classified by KOG to be involved in lipid transport and metabolism. To validate the microarray results, we performed quantitative RT-PCR on a subset of four genes found to be altered in expression; three from the lipid transport and metabolism KOG category, and one from the carbohydrate metabolism category. At 22°C, the null mutant strain failed to upregulate the expression of a lipid transfer protein and acetoacetyl-CoA synthase (**Figure 8**). Conversely, the expression of a peroxisomal dehydratase was over-expressed at 37°C and 22°C, when compared to the wild-type isolate (**Figure 8**). We also confirmed the altered expression of a glycosyl hydrolase postulated to be involved in cell-wall remodeling. In the null mutant, this gene is over-expressed at 37°C and 22°C, when compared to the wild-type isolate (**Figure 8**). Thus, we have begun to identify candidate genes and processes that may be direct or indirect targets of *SREB* and contribute to the phase transition from yeast to mold.

## Discussion

The use of *A. tumefaciens*-mediated DNA transfer for insertional mutagenesis has advanced our understanding of the endemic dimorphic fungi at the molecular level [10],[12],[13],[28]. We used this technology to mutate *B. dermatitidis* conidia and screen for transformants with altered morphology during growth at 22°C and 37°C. Analysis of mutant 3-15-1 uncovered a GATA transcription factor, *SREB*, which regulates siderophore biosynthesis and affects morphology in *B. dermatitidis*. GATA transcription factors are zinc-finger proteins that bind conserved motifs to induce or repress gene expression [16],[22],[26],[27],[29]. These genes are found widely in eukaryotes, but they function differently in fungi, plants, and animals [30],[31]. In fungi, GATA transcription factors regulate diverse functions including the response to blue light, switching of mating-type, uptake of nitrogen, pseudohyphal growth during nitrogen starvation, biosynthesis of siderophores, and iron assimilation [17],[22],[29],[32],[33].

Our analysis indicates that *SREB* has pleotropic effects in *B. dermatitidis* - it promotes the transition from yeast to mold at environmental temperature and represses the biosynthesis of siderophores. Following a shift of incubation temperature from 37°C to 22°C, the insertional and null mutants were unable to complete the phase transition or accumulate significant biomass when compared to the parent strain. To our knowledge, *B. dermatitidis SREB* is the first gene identified in the dimorphic fungi that promotes the conversion of yeast to mold. Much of the field's attention has been focused on genes that regulate the phase transition from mold to yeast; only a few genes have been identified that regulate growth or morphology in the dimorphic fungi at environmental temperature (i.e. 22–25°C). In *H. capsulatum*, the mold-specific gene *MS8* regulates mycelial morphology and growth, but not the phase transition [34]. In *P. marneffei TupA* is required for maintenance of mycelial morphology at 25°C; null mutants convert to mycelia following a temperature shift from 37°C to 25°C, but revert to yeast morphology with prolonged incubation [35].

We hypothesize that *B. dermatitidis SREB* binds DNA to regulate many genes that, in turn, control such disparate functions as phase transition and the response to abiotic stress, including iron availability. Using MAST analysis we identified a large number of genes with putative GATA transcription factor binding sites. When compared to the entire *B. dermatitidis* genome, candidate genes involved with the biosynthesis of secondary metabolites as well as amino acid and lipid metabolism were found to be over-represented. Some of these candidate genes were indeed altered in expression in *SREB*Δ, as detected in preliminary microarray analysis and validated by RT-PCR. The enrichment of genes involved in secondary metabolism and amino acid metabolism were not unexpected, in part, because *SREB* regulates siderophore biosynthesis, a process that requires the transport and metabolism of amino acids. The abundance of genes containing GATA binding sites involved in lipid transport and metabolism was surprising. To our knowledge, regulation of lipid metabolism and transport in fungi by GATA transcription factors has not been described.

Changes in fatty acid metabolism in the dimorphic fungi are associated with the phase transition and are postulated to impact morphogenesis [36]–[42]. Exposure of *H. capsulatum* mycelia to unsaturated fatty acids prolongs the mold-to-yeast conversion following a shift in temperature from 25 to 37°C [36]. In contrast, treatment with saturated fatty acids accelerates the phase transition [36]. In *C. immitis*, exposure to exogenous fatty acids alters the conversion of spherules to mycelia [37]. Reduced expression of the Δ^9^-desaturase gene, *OLE1*, in *C. albicans*, impairs hyphal formation [38]. Differences in the concentration of unsaturated fatty acids (oleic and linoleic acids) and unsaturated sphingolipids (*N*-2′-hydroxy-(*E*)-Δ^3^-octadecenoate) have been described in the yeast and mold forms of *H. capsulatum* and *P. brasiliensis* [39]–[42]. In *P. brasiliensis*, several genes involved in lipid metabolism, have been demonstrated to be phase-regulated [43]. Thus, further investigation of genes involved in fatty acid metabolism may clarify the mechanism by which *SREB* promotes the phase transition from yeast to mold.

*B. dermatitidis* insertional and null mutants have multiple alterations in the regulation of iron assimilation, as indicated by their yellow-orange appearance, constitutive production of siderophores, and derepression of iron-regulated genes during conditions of iron abundance.

Iron acquisition must be tightly regulated for proper cellular function and to avoid toxicity due to iron overload [17],[44]. Under iron-replete conditions, *SREB* represses genes involved in the production (*SIDA, AMCA*) and uptake (*MIRB, MIRC*) of siderophores. *AMCA* encodes a transferase that shuttles ornithine from the mitochondria to the cytosol [44]. The first step in siderophore biosynthesis involves the conversion of ornithine into N^5^-hydroxy-L-ornithine, which is catalyzed by an L-ornithine-N^5^-monooxygenase encoded by *SIDA* [45]. Siderophores secreted into the environment bind iron and then can be taken up by the cell through permeases such as *MIRB* and *MIRC* [46]. Analysis of the *B. dermatitidis* genome did not reveal an ortholog to *A. nidulans MIRA*, which facilitates the uptake of xenosiderophores, specifically enterobactin [46]. Deletion of *SREB* resulted in de-repression of *SIDA, AMCA, MIRB,* and *MIRC* expression under iron-replete conditions. Similar to *P. chrysogenum SREP*, *N. crassa SRE*, and *A. nidulans SREA* null mutants, disruption of *SREB* in *B. dermatitidis* resulted in discoloration of the fungus [14],[18],[45]. In addition, we identified two extracellular siderophores, dimerum acid and coprogen, produced by *B. dermatitidis* when grown under iron-poor conditions. When iron is abundant, *SREB* represses the biosynthesis of both these siderophores.

Similar to *A. nidulans* and *H. capsulatum*, the expression of *B. dermatitidis SREB* is upregulated when iron is abundant, and repressed when iron is limited [16],[17]. Repressors of siderophore biosynthesis are not uniformly regulated at the transcriptional level in other fungi, as orthologs of *SREB* including *SRE, URBS1, FEP1, and SFU1* are constitutively expressed regardless of exogenous iron concentrations [18]–[21]. *SREB* is expressed as a single transcript, similar to *SRE* and *URBS1* [18],[19]. In contrast, *SREP*, *SREA*, and *FEP1* are expressed as two separate transcripts due to the presence of two transcriptional start sites [14],[17],[20].

*B. dermatitidis SREB* may participate in a regulatory circuit with the bZIP (basic leucine zipper) transcription factor, *HAPX*. Computational analysis of the promoter region of *HAPX* in *B. dermatitidis* revealed putative GATA binding sites. Moreover, iron-poor conditions induced *HAPX* expression in wild-type *B. dermatitidis*, whereas iron abundance reduced its expression. In *A. nidulans*, *HAPX* represses *SREA* as well as genes that encode iron-dependent proteins such as *CYCA* (cytochrome C), *ACOA* (aconitase), *LYSF* (homoaconitase) when iron availability is limited [44]. We found that deletion of *SREB* resulted in the expression of *HAPX* under iron-poor and iron-replete conditions.

Our findings support the idea that *B. dermatitidis SREB* functions as a transcription factor that regulates the biosynthesis of siderophores and promotes the conversion from yeast to mold. We propose that *SREB* inhibits genes involved with the biosynthesis and uptake of siderophores under conditions of iron abundance. Our findings also suggest that *SREB* affects phase transition independently of iron assimilation, perhaps, by altering the expression of genes involved with lipid metabolism or cell wall remodeling. The iron-related defects do not explain the failure to convert from yeast to mold since growth under iron-poor conditions had no effect on the defect in morphogenesis. GATA transcription factors in other fungi have been demonstrated to regulate morphogenesis as well as the response to temperature. *S. cerevisiae ASH1* encodes a GATA transcription factor that inhibits mating-type switching and induces filamentous growth under conditions of nitrogen limitation [29]. *C. neoformans CIR1*, an ortholog of *B. dermatitidis SREB*, regulates genes involved in reductive iron assimilation and siderophore transport, but also genes critical for virulence including those required for thermotolerance, capsule production, and melanin biosynthesis [22].

In summary, we identified and characterized a GATA transcription factor that represses the biosynthesis of siderophores and promotes the phase transition from yeast to mold. To our knowledge, *B. dermatitidis SREB* is the first gene identified in dimorphic fungi that promotes the conversion of yeast to mycelia. By using bioinformatic and expression analyses we identified several genes whose expression may be directly or indirectly regulated by *SREB*. We investigated a sample of these genes, including ones in KOG categories for lipid and carbohydrate metabolism, and found that their expression is affected by the deletion of *SREB*. Future work will strive for a more complete description of how *SREB* promotes the yeast to mold phase transition. Because growth in the mold form is thought to be essential for the survival of dimorphic fungi in nature and the generation of infectious particles, *SREB* may be needed for the evolutionary maintenance of this species. The generation of an *SREB* null mutant provides a unique opportunity to elucidate the *SREB* regulon and identify genes that govern growth in the mold form, as well as other traits in this human fungal pathogen.

## Materials and Methods

### Strains and growth conditions

*Blastomyces dermatitidis* strains used in this study included T53-19 and American Type Culture Collection (ATCC) 26199. T53-19 sporulates, but is weakly virulent in a murine model of infection, and ATCC strain 26199 is highly virulent, but does not sporulate [10],[47]. The genome of strain 26199 has been sequenced by the Genome Sequencing Center at Washington University (http://genome.wustl.edu). *B. dermatitidis* yeast and mold were grown on *Histoplasma* macrophage medium (HMM), 3M medium (3M), Potato dextrose agar (PDA), or Middlebrook 7H10 agar medium containing oleic acid-albumin complex (7H10; Becton Dickinson and Company, Franklin Lakes, NJ) [48]–[50]. *Agrobacterium tumefaciens* strain LBA1100 harboring the Ti helper plasmid pAL1100 (gift from C. van den Hondel; Leiden University, The Netherlands) was maintained on Luria-Bertani (LB) medium supplemented with 0.1% glucose, spectinomycin 100 µg/ml, and kanamycin 100 µg/ml once transformed with a binary vector [28].

### Insertional mutagenesis

Conidia from *B. dermatitidis* strain T53-19 were mutagenized using *A. tumefaciens* containing pBTS165 [10],[28],[51]. This binary vector contains a resistance cassette, hygromycin phosphotransferase (*hph*), integrated into the T-DNA that is driven by a glyceraldehyde-3-phosphate dehydrogenase (*gpdA*) promoter derived from *Aspergillus nidulans* [10]. Conidia harvested from mycelial cultures by manual disruption were counted using a hemocytometer, suspended in phosphate buffered saline (PBS) to a final concentration of 2×10^7^/ml, and co-cultivated with *A. tumefaciens* (6×10^8^ cells/ml) on a Biodyne A nylon membrane (Pall Gelman, Ann Arbor, MI) on induction medium containing 200 µM acetosyringone (IMAS medium) [28]. After 72 hours of incubation at 22°C, the biodyne membranes were transferred to 3M medium supplemented with hygromycin 100 µg/ml (AG Scientific Inc., San Diego, CA) and cefotaxime 200 µM (Sigma-Aldrich), and incubated at 37°C or 22°C. Individual transformants were visually screened by light microscopy for altered morphology: growth as hyphae or pseudohyphae at 37°C or yeast at 22°C. Replica plates were used to identify transformants that lost viability upon shifting the incubation temperature from 22°C to 37°C.

### Adaptor PCR

Adaptor PCR was used to amplify DNA flanking the pBTS165 insert from insertional mutant 3-15-1 [52]. Following the digestion of genomic DNA by restriction enzymes *Stu*I, *Hpa*I, and *Xmn*I, which do not cut in pBTS165, adaptors were ligated to the restriction fragments using T_4_ DNA ligase (New England Biolabs, Ipswich, MA). PCR was performed using primers specific for the adaptors and pBTS165. The PCR products were separated by agarose gel electrophoresis and purified using the QIAquick gel extraction kit (Qiagen, Valencia, CA) and sequenced by the DNA Sequencing Laboratory at the University of Wisconsin Biotechnology Center. Sequence flanking the insert was analyzed using GSC (Genome Sequencing Center) BLAST (http://genome.wustl.edu/tools/blast) and National Center for Biotechnology Information (NCBI) tBLASTx (http://blast.ncbi.nlm.nih.gov/Blast.cgi). FGENESH was used to identify predicted exons and introns in the *SREB* gene (www.softberry.com).

### Generation of null mutants

Two vectors, pBTS4-KO1 and pBTS4-KO2, were used to delete *SREB* in *B. dermatitidis* strain 26199 by homologous recombination and resulted in two null mutants, T1#23 and T12#16, respectively. Although both null mutants had similar phenotypes, T1#23 contained an additional 2,214 bp deletion in the 5′ untranslated region that was upstream of the disrupted *SREB* gene. Herein, T12#16, which has no additional deletions, is referred to as *SREB*Δ. Plasmid pBTS4-KO2 contained 1611 bp of 5′ upstream sequence and 1747 bp of coding and 3′ downstream sequence flanking *hph*. The 1611 bp and 1747 bp products were amplified from *B. dermatitidis* 26199 genomic DNA using F and R primers containing *Sac*I, *Bbs*I, *Sbf*I, or *Cla*I restriction sites (F-1611-*Sac*I 5′-TTTGAGCTCACTTTACTCTTCGGACGGGTTTT; R-1611-*Bbs*I 5′-TTTTCGATTGTCTTCAGCCAAAAGCCCCGTCATTCCTGT; F-1747-*Sbf*I 5′-TT-TCCTGCAGGTTGCAGCGTGAGGCGGAAGA; R-1747-*Cla*I 5′-TTTATCGATTGACAGGGCAG-GCTACATA). PCR products were separated by agarose gel electrophoresis, purified using QIAquick PCR purification kit (Qiagen, Valencia, CA), sequenced, and ligated into pBTS4 in sequential fashion following restriction digest to flank the *hph*-resistance cassette [53]. After sequence and restriction digest analyses confirmed integration of the ligated PCR fragments, pBTS4-KO2 was electroporated into *A. tumefaciens* strain LBA1100 [28]. *B. dermatitidis* strain 26199 (2×10^7^ yeast/ml) was transformed with *A. tumefaciens* containing pBTS4-KO2 (6×10^8^ bacteria/ml) on Biodyne A membranes on IMAS medium. After 72 hours of incubation at 22°C, the Biodyne membranes were transferred to HMM medium supplemented with 10–20 µM FeSO_4_, hygromycin 25 µg/ml, cefotaxime 200 µM, and incubated at 37°C. Transformants were visually screened for yellow pigmentation. The null mutant was cloned to obtain individual colonies and establish a line of cells. *SREB* gene deletion was confirmed by PCR, and Southern and Northern blot analyses (see below).

### Complementation of insertional and null mutants

Insertional mutant 3-15-1 was re-transformed with pBTS47-11+13 using *A. tumefaciens*-mediated DNA transfer. This plasmid contained the *SREB* coding region, 1990 bp of 5′ sequence upstream of the start codon, 603 bp of 3′ sequence downstream of the stop codon, and a nourseothricin resistant cassette. Genomic DNA was amplified using primers ggp11-*Xba*I (5′-TTTCTAGAACAACTACCTCTACATGACACT-GC) and ggp13-*Sbf*I (5′-TTTCCTGCAGGGAGCCTTTTCTTTCTGTCAA). The PCR products were separated by agarose gel electrophoresis, purified using QIAquick PCR gel extraction kit (Qiagen, Valencia, CA), sequenced, and ligated into pBTS47 to generate pBTS47-11+13. The null mutant, *SREB*Δ, was re-transformed by *A. tumefaciens* with pBTS47-5331, which contains the *SREB* coding region, 2655 bp of 5′ sequence upstream of the start codon, 603 bp of 3′ sequence downstream of the stop codon, and a nourseothricin resistant cassette. The protocol for *A. tumefaciens*-mediated DNA transfer was similar to that described in the previous section. Transformants were screened for white colony pigmentation on HMM medium supplemented with 20 µM FeSO_4_, nourseothricin 25 ug/ml (Werner Bioagents, Germany), and cefotaxime 200 µM at 37°C incubation.

### DNA extraction & Southern blot hybridization

*B. dermatitidis* was grown to late log phase in liquid HMM at 37°C incubation. Genomic DNA was extracted using the method described by Hogan and Klein [54]. Southern blot hybridization was performed as described [28],[55]. The fate of the transforming DNA in the insertional mutant was determined using probes specific for T-DNA and non-T-DNA sequences. An 822 bp amplicon constructed using primers 5′-CGATG-TAGGAGGGCGTGGATA and 5′-GCTTCTGCGGGCGATTTGTGT was used to probe *hph* within the T-DNA. An 8 kb *Bgl*II restriction fragment generated from pBTS4 was used to probe the non-T-DNA sequence. Deletion of *SREB* in the null mutant was analyzed using PCR-generated probes specific for the *SREB* coding region (1303 bp; 5′-CCCGCTCTTTGCTTAACC-CGTATG and 5′-CTGGTGATAAAGAAGGGCTGAA), *hph* (822 bp; 5′-CGATGTAGGAGGGCG-TGGATA and 5′-GCTTCTGCGGGCGATTTGTGT), 5′ region flanking *SREB* (1663 bp; 5′-ACTT-TACTCTTCGGACGGGTTTTC and TATCTGCGCTTTTGGTAGTAGGAG), and the 3′ region flanking *SREB* (1747 bp; 5′-TTGCAGCGTGAGGCGGAAGA and 5′-ACAAATCGTAGCACCAG-TC). All probes were radiolabeled with α-^32^P dCTP using a Prime-a-Gene labeling system (Promega, Madison, WI). Unincorporated radionucleotides were removed using ProbeQuant G50-micro columns (GE Healthcare, Buckinghamshire, UK). Following hybridization, the blot was washed sequentially with low stringency (0.25 M NaPO_4_, 2% SDS, 1 mM EDTA) and high stringency (0.04 M NaPO_4_, 1% SDS, 1 mM EDTA) solutions, exposed to a storage phosphor screen (Molecular Dynamics, Sunnyvale, CA) and scanned using a Storm 660 imaging system (Molecular Dynamics, Sunnyvale, CA).

### Ferric perchlorate assay

Ferric perchlorate was used to measure siderophore production semi-quantitatively [25]. *B. dermatitidis* was grown at 37°C in liquid 3M or HMM under iron-poor or replete (10 µM FeSO_4_) conditions. Iron-poor media consisted of HMM or 3M prepared with F-12 Ham's nutrient mixture lacking FeSO_4_, or trace elements lacking FeSO_4_, respectively. In addition, exogenous iron was not added to these media. As the yeast entered stationary growth (A_600_ = 3.5−4.0), culture supernatants were collected, filtered (0.2 µM), and added to a ferric perchlorate solution (5 mM Fe(ClO_4_)_3_ in 0.1 N HCl). Absorbance was measured at 425 or 495 nm. Plasticware was used whenever possible. Glassware was treated with 2N HCl to remove residual traces of iron [56]. Analysis of variance (ANOVA) was used to analyze the results from the ferric perchlorate assay. Tukey's Honest Significant Difference method was used to adjust the p-values for multiple comparisons.

### RNA extraction & Northern blot hybridization

*B. dermatitidis* was grown to mid-log phase at 37°C in liquid HMM with no added iron (iron-poor medium), 10 µM FeSO_4_, or 50 µM FeSO_4_. Total RNA was extracted using the phenol-guanidinium thiocyanate-1-bromo-3-chloropropane extraction method [55]. In brief, yeast were washed with PBS, beaten with beads, and treated with TRI Reagent followed by 1-bromo-3-chloropropane (Molecular Research Center Inc., Cincinnati, OH). RNA was precipitated using a 1∶1 concentration of isopropanol and a high salt solution (Molecular Research Center Inc., Cincinnati, OH), washed with 75% ethanol, and resuspended in water that was pre-treated with diethyl pyrocarbonate (DEPC; Calbiochem, San Diego, CA). Total RNA was further purified using RNeasy kit (Qiagen, Valencia, CA) and enriched for mRNA using oligo(dT)-polystrene chromatography (Sigma-Aldrich). Northern hybridization was performed as described using 2.0-2.3 µg poly(A)^+^-enriched mRNA per sample [55]. Gene expression was analyzed using probes constructed by PCR against *SREB* (SreF 5′-CCCGCTCTTTGCTTAACCCGTATG; SreR 5′-CTGGTGATAAAGAAGGGCTGAA) *SIDA* (SidA-F1 5′-AGACAGTACTCAAGAACGACAA; SidA-R1 5′-GCTGTCATCGCTGGGCTTTAGTGC), *MIRB* (MirB-F 5′-CTCCTCCTCGTCGCTTTCGCACTA; MirB-R 5′-CCCTGAGGTCCCCGT-AGATGAG), *MIRC* (MirC-F 5′-TGATGGCATTCTCAACCTCCC; MirC-R 5′-AACCTGCGGTGAT-GAAACCAC), *AMCA* (AmcA-F 5′-GTCCGCATTACTCATCTG; AmcA-R 5′-CGCCTCATAAATC-GTAA), HAPX (HapX-F 5′-CCGGTACCCCTCAAGCCCACAACT; HapX-R 5′-AAATACTTCAAC-ACGCCCATAACG), and actin (Actin-F 5′-TCGGCCGTCCTCGCCATC and Actin-R 5′-TCCAG-ACTCGTCGTAGTCCTGC).

### Real-time PCR

Total RNA was extracted from *B. dermatitidis* wild-type and *SREB* null mutant strains grown in HMM at 37°C and 22°C in a similar fashion as described above; modifications included grinding cells frozen in liquid nitrogen in a mortar and pestle. Wild-type and *SREB* null mutant cells were grown for 48 hours at 22°C prior to RNA extraction. RNA, at 10 ug/sample, was treated with Turbo DNase (Applied Biosystems/Ambion, Austin, Tx) and further purified using RNeasy kit (Qiagen, Valencia, CA). cDNA was generated from 1 ug of DNase-treated RNA using iScript cDNA synthesis kit (Bio-Rad, Inc., Hercules, CA). Real-time PCR reactions were comprised of 1x SSoFast EvagGreen supermix (Bio-Rad), 0.5 mM of each primer, and 1 ul of 10-fold diluted cDNA template in a total volume of 10 ul. All reactions were performed in triplicate for two biological replicates. Real-time PCR was performed using a Bio-Rad iCycler MyiQ. Cycling conditions were 1 cycle at 95°C for 30 seconds followed by 40 cycles of 95°C for 5 seconds and 60°C for 10 seconds. Melting curve analysis was performed following the completion of the PCR. Gene expression was normalized relative to the expression of alpha-tubulin based on R (relative expression) = 2^−ΔCt^, ΔCt = Ct_target gene_–Ct_tubulin_ [57]. Primers used to amplify transcripts from the following genes were: Lipid transfer protein (BDBG_03618-1F 5′- CCATCAATGCTGCCATCAAC; BDBG_03618-1R 5′-GGTCTCACCCTTGTCGTTTG), glycosyl hydrolase (BDBG 03183-1F 5′-GCTCTCCCAAGACATACATCAG, BDBG_03183-1R 5′-CCAT-AGCAAACTTCCCAAAAG), peroxisomal dehydratase (BDBG_00052-1F 5′-CCCATTGTGCTA-ACCTTCAAG, BDBG_00052-1R 5′-AACTCCATCCGTCGCCTC), acetoacetyl-CoA synthase (BDBG_09522-1F 5′-GCTCTCGGCACGCTCATAC, BDBG_09522-1R 5′-GGTGGTGACGG-GAGAAATG) and alpha-tubulin (BDBG_00020-2F 5′-GGTCACTACACCATCGGAAAG-3′, BDBG_00020 2R 5′-CTGGAGGGACGAACAGTTG).

### MAST (motif alignment and search tool) analysis and KOG (eukaryotic orthologous groups) annotation

The annotated genome and predicted proteome of *B. dermatitidis* strain SLH14081 was used for MAST analysis and KOG annotation. The genome of this strain (75.35 Mb; 9,555 genes) has been sequenced and annotated by the Broad Institute (www.broadinstitute.org/annotation/genome/blastomyces_dermatitidis/MultiHome.html and ACBT01000000). The absence of annotation in the sequenced genome of 26199 precluded its use for computational analysis.

MAST/MEME (multiple em for motif elicitation) software in unix (version 4.2.0) was used to identify GATA transcription factor binding motifs in the genome of *B. dermatitidis* SLH14081 [58]. A fifth-order Markov background model was built for SLH14081 using the MEME utility fasta-get-markov. To find the location of previously identified motifs, MAST was run with a given motif frequency table, the Markov background model (-bfile) and options to produce text output as a ‘hit list’ (–text –hit_list). For a search with the ATCwgAtaa motif [26], a p-value of 0.0005 was used (–mt 0.0005). MAST output and Broad gene coordinates (http://www.broadinstitute.org/annotation/genome/blastomyces_dermatitidis/MultiHome.html) were parsed using a custom perl script to find intergenic motifs <2kb upstream of predicted genes. A total of 84,965 motifs were found in the genome assembly, of which 79,458 were in intergenic regions. Of these, 3,372 copies were found <2 kb upstream of 2,468 genes. Genes with the following motifs were retained: ATC-(A/T)-GATA-(A/G), ATC-(A/T)-GATA-(T/C), ATC-(A/T)-GATT-A, ATC-(A/T)-GATC-A, ATC-A-GATG-A, ATC-C-GATA-A, and ATC-A-AATA-A. These motifs are found upstream of genes regulated by *A. fumigatus SREA*, an *SREB* homolog [26].

To discover new motifs using MEME, we identified orthologs in SLH14081 of the iron-upregulated genes from *A. fumigatus* (BDBG_00046, BDBG_00047, BDBG_00048, BDBG_00050, BDBG_00053, BDBG_00054, BDBG_00055, BDBG_01314, BDBG_02226, BDBG_06775, BDBG_06965, BDBG_08034, BDBG_08208, BDBG_09322) and searched the 1 kb upstream for common motifs; MEME options were set for any number of motifs per region (-mod anr), the above described Markov background model, and a minimum width of 6 (-minw 6). This identified a motif of vATCwGATAA, which is similar to the motif described by Hwang and colleagues [27].

For KOG annotation and analysis, the predicted proteome from *B. dermatitidis* strain SLH14081 was retrieved from the Broad institute (http://www.broadinstitute.org/annotation/genome/blastomyces_dermatitidis/MultiDownloads.html, accessed: 11/09/2009) and compared against the NCBI KOG database (ftp://ftp.ncbi.nih.gov/pub/mmdb/cdd/, accessed: 11/09/2009)) using RPSBLAST (e-value 1e-05) [59],[60]. Two data sets were generated with the first containing all *B. dermatitidis* genes encoding proteins that registered a KOG annotation. The second set included *B. dermatitidis* proteins encoded by the candidate genes with upstream GATA sites. The KOGs for both sets were correlated to their associated categories, and the total number of proteins within each category was tabulated. A two-tailed Fisher's exact test was used to determine if the number of proteins in each category were over- or under-represented when compared to all KOG-annotated proteins in the *B. dermatitidis* proteome. Categories were considered over-represented if the p-value of the right of the Fisher's exact test was less than 0.05 and over-represented if the left tail was less than 0.05.

### Isolation & identification of siderophores

To isolate and identify siderophores produced by *B. dermatitidis* 26199 wild-type and null mutants, we used column chromatography, liquid chromatography/mass spectroscopy (LC/MS), and reverse-phase high-pressure liquid chromatography (HPLC). Supernatants were harvested from *B. dermatitidis* grown in liquid HMM at 37°C under iron-poor (no added iron) and iron-replete (10 µM FeSO_4_) conditions when the cultures entered stationary growth (A_600_ = 3.5−4.0). Culture supernatants were filtered (0.2 µM), treated with 2% ferric chloride and applied to a column (K 9/30, GE Healthcare) packed with Amberlite XAD-2 resin (Supelco, Bellefonte, PA). The resin and column were prepared according to the manufacturer's recommendations. Following a water wash (7 bed volumes; flow rate of 0.2 ml/min), siderophores were eluted from the resin using methanol (1.7 bed volume; flow rate of 0.1 ml/min), reduced to dryness, and re-suspended in water (100 µl). Colorless supernatants that contained siderophores developed an orange color when treated with ferric chloride. This allowed for visual assessment of binding and elution of siderophores from the resin [61],[62].

The Mass Spectroscopy Facility at the University of Wisconsin Biotechnology Center performed LC/MS analysis of concentrated eluate collected from wild-type *B. dermatitidis* grown under iron-poor conditions following XAD-2 column chromatography. For HPLC, siderophores were separated on a C18 column (Agilent Eclipse XDB-C18 column; 4.6×150 mm) using a water-acetonitrile gradient containing 0.1% trifluoroacetic acid (Sigma-Aldrich). The gradient of acetonitrile was increased from 5% to 15% over 15 minutes, and 15% to 25% over 35 minutes. The flow rate was 0.5 ml/min and the absorbance was measured at 465 nm. Retention times were compared to siderophore standards (HPLC calibration kit – coprogens and fusarinines; EMC microcollections, Tubingen, Germany).

### Accession numbers

The nucleotide sequences for *SREB*, *SIDA, AMCA, MIRB, MIRC,* and *HAPX* from *B. dermatitidis* strain 26199 were obtained from the Genome Sequencing Center, Washington University, Saint Louis, MO (http://genome.wustl.edu/tools/blast). Although this genome is publically available, it is not annotated. Allelic sequences can be found at the Broad Institute (http://www.broadinstitute.org/annotation/genome/blastomyces_dermatitidis/MultiHome.html) and have the following gene locus identification numbers: *SREB* (BDBG_01059), *SIDA* (BDBG_00053), *AMCA* (BDBG_00128), *MIRB* (BDBG_05798), *MIRC* (BDBG_08034), *HAPX* (BDBG_01314). Additional gene locus numbers include: lipid transfer protein (BDBG_03618), glycosyl hydrolase (BDBG_03183), peroxisomal dehydratase (BDBG_00052), acetoacetyl-CoA synthase (BDBG_09522), and alpha-tubulin (BDBG_00020).

## Supporting Information

Figure S1 Insert analysis of insertional mutant 3-15-1. Southern blot analysis of *Sac*I digested genomic DNA of 3-15-1 and parental strain T53-19 probed against hygromycin resistance cassette (A) and non-T-DNA vector sequences (B). Both probes hybridized to a single genomic fragment of 11.5 kb, indicating that the whole pBTS165 plasmid has been transferred and inserted into a single site in the genome of mutant 3-15-1 (arrows). Bands in the parent and mutant other than 11.5 kb represent non-specific binding by both probes.
(0.56 MB EPS)

Table S1 KOG-annotation of *B. dermatitidis* genes with GATA sites identified by MAST analysis. KOG annotated genes containing GATA motifs (ATC-A/T-GATA-A/G, ATC-A/T-GATA-T/C, ATC-A/T-GATT-A, ATC-A/T-GATC-A, ATC-A-GATG-A, ATC-C-GATA-A, and ATC-A-AATA-A) located less than 2 kb upstream are organized according to KOG classification. From the MAST analysis, the gene locus, gene description, DNA strand (+/-), score, p-value, sequence, and location of the upstream GATA motif are listed. KOG identification (KOG ID) number is provided for each KOG-annotated gene.
(0.15 MB XLS)
